# Hybrid eclipse: a case of isolated renal tuberculosis with an unusual infectious–inflammatory–neoplastic triad

**DOI:** 10.3389/fmed.2026.1777156

**Published:** 2026-07-17

**Authors:** Sachin Dharwadkar, Vijaykumar Ramu, Kavitha B. Chittaragi, Suchitha Sathish, Sudha Kiran Das, Sajan Sehgal

**Affiliations:** 1 Department of Urology, JSS Medical College, JSSAHER, Mysuru, India; 2 Department of Radiology, JSS Medical College, JSSAHER, Mysuru, India; 3 Department of Pathology, JSS Medical College, JSSAHER, Mysuru, India

**Keywords:** computed tomography, nephrocutaneous fistula, renal cell carcinoma, renal replacement lipomatosis, renal tuberculosis

## Abstract

Isolated renal tuberculosis (RTB) is a rare extrapulmonary manifestation of tuberculosis that often mimics common urinary tract infections, resulting in delayed diagnosis and complications. We report an exceptionally rare case of isolated RTB presenting with a spectrum of destructive sequelae—including a perinephric abscess with a nephrocutaneous fistula, renal replacement lipomatosis (RRL), and synchronous clear cell renal cell carcinoma (RCC) within a single kidney. We report the case of a 77-year-old diabetic man who presented with left loin pain and chronic lumbar sinus discharge. Multiphase computed tomography (CT) revealed an enlarged, distorted kidney with extensive sinus and perinephric fat proliferation consistent with RRL; a hyperenhancing superior-pole mass suggestive of RCC; calyceal distortion and infundibular stenosis suspicious for RTB; and a perinephric abscess with a cutaneous fistula. Radical nephrectomy was performed, and histopathological examination confirmed renal tuberculosis and its associated complications. Subsequent antitubercular therapy (ATT) resulted in stable clinical and functional outcomes. To the best of our knowledge, this is the first reported case demonstrating this constellation of infectious–inflammatory–neoplastic pathologies within a single kidney. This case highlights the indolent yet destructive nature of isolated RTB and emphasizes the role of early imaging and histopathology for timely diagnosis, prevention of serious complications, and an improved prognosis.

## Introduction

Isolated renal tuberculosis is a rare form of extrapulmonary tuberculosis that occurs without pulmonary involvement and often presents with non-specific symptoms, resulting in delayed diagnosis (1).

The rising global burden of tuberculosis, particularly in developing countries such as India, has led to a proportional increase in extrapulmonary forms. This trend is further compounded by risk factors including human immunodeficiency virus infection, immunosuppressive states, malnutrition, and underlying metabolic conditions, such as diabetes mellitus. Urogenital tuberculosis accounts for approximately 27% of extrapulmonary tuberculosis cases, ranking after pleural and lymphatic involvement (2, 3). Renal involvement most often results from hematogenous dissemination of pulmonary tuberculosis. Isolated renal disease is rare and may arise from ascending genitourinary infection or delayed hematogenous seeding from a healed pulmonary focus (4).

This report describes an exceptionally rare and complex case of isolated renal tuberculosis manifesting with a wide spectrum of complications, including chronic pyelonephritis, a perinephric abscess with a nephrocutaneous fistula, renal replacement lipomatosis (RRL), and synchronous clear cell renal cell carcinoma (RCC) within a single kidney. The diagnostic complexity and extensive renal destruction necessitated a nephrectomy, and postoperative histopathology established the definitive diagnosis of renal tuberculosis, following which the patient was initiated on antitubercular therapy (ATT). The coexistence of infectious, inflammatory, degenerative, and neoplastic processes within a single kidney is extraordinarily uncommon and, to the best of our knowledge, has not been previously reported.

This case highlights the silent progression of renal tuberculosis and its coexistence with a rare and complex spectrum of pathological processes, including malignancy, thereby underscoring the importance of early diagnosis and timely treatment to prevent irreversible renal damage and avoid nephrectomy.

## Case report

We report the case of a 77-year-old man who presented with a 10-day history of dull, aching pain in the left loin associated with intermittent serous discharge from the left lumbar region. His medical history was significant for type 2 diabetes mellitus and hypertension, for which he was receiving regular medical treatment. He also had a history of renal calculus disease and recurrent urinary tract infections.

He had no prior history of tuberculosis, constitutional symptoms such as weight loss, or other chronic systemic illnesses. No additional urinary or respiratory symptoms were reported at presentation.

### Diagnostic assessment

Physical examination revealed tenderness and induration at the left renal angle with active serous discharge, but no fever or systemic signs. Laboratory evaluation showed mild anemia (hemoglobin [Hb]: 11.2 g/dL) and mildly elevated serum creatinine (1.3 mg/dL). Urinalysis showed pyuria and hematuria. Two early morning urine specimens were analyzed for acid-fast bacilli (AFB) by microscopy, and both were negative. A clean-catch midstream urine sample was submitted for aerobic bacterial and fungal cultures according to institutional laboratory protocol. No bacterial growth was observed after 48 h of incubation, and fungal cultures remained negative after 28 days. GeneXpert testing for *Mycobacterium tuberculosis* was also negative.

Chest radiograph was normal. Ultrasonography showed an enlarged, distorted left kidney with lower-pole calculi. Multiphase contrast-enhanced computed tomography (CT) revealed an enlarged, distorted left kidney with marked asymmetric parenchymal thinning and lower pole calculi. Extensive renal sinus, hilar, and perinephric fat proliferation was noted, distorting the pelvicalyceal system, which is consistent with renal replacement lipomatosis. CT urography images demonstrated calyceal distortion, infundibular stenosis, and pelvic wall thickening, suspicious for renal tuberculosis (Figure 1). A large peripherally enhancing perinephric collection (10.0 × 6.4 × 7.5 cm) was found to extend into the left psoas muscle and posterior abdominal wall, forming a nephrocutaneous fistulous tract. Additionally, a well-defined hyperenhancing superior-pole mass (3.1 × 2.9 cm) was identified, suggestive of malignancy (Figure 2). Perinephric fat stranding was also observed, indicating pyelonephritis.

**Figure 1:**
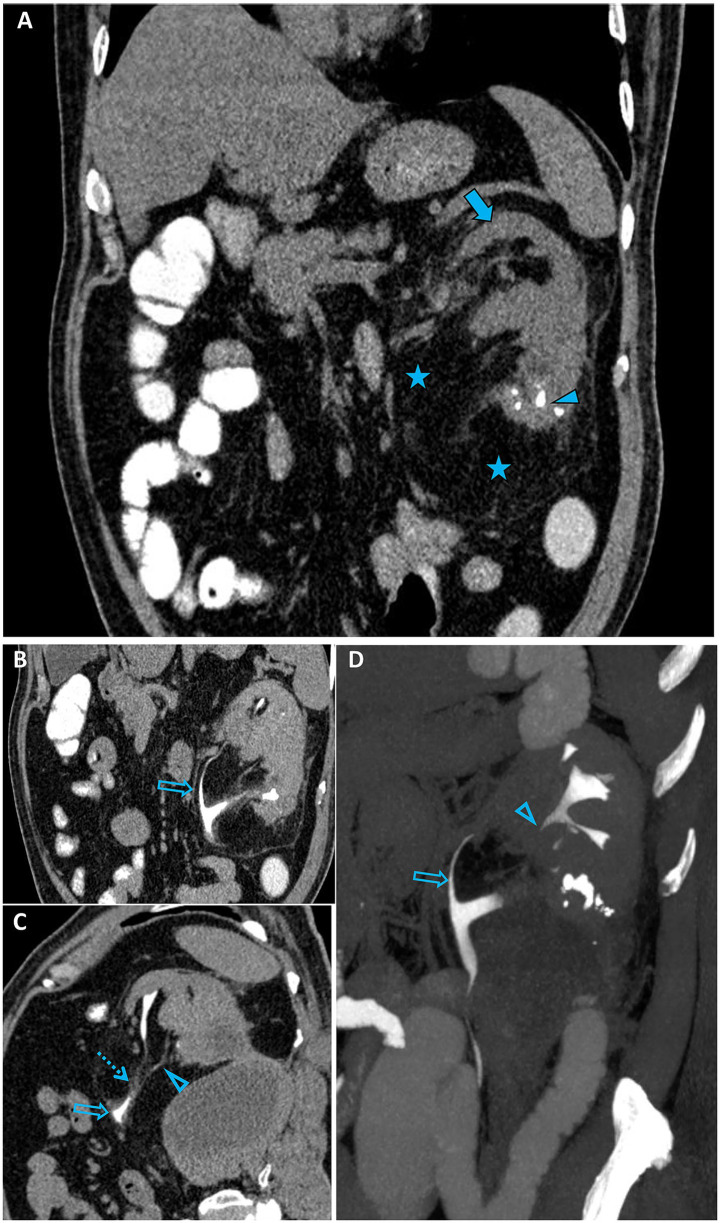
Non-contrast CT and urography findings. **(A)** Coronal non-contrast CT of the abdomen demonstrates an enlarged left kidney with asymmetric parenchymal thinning in the upper pole (solid arrow), lower-pole parenchymal calcifications (solid arrowhead), and extensive fat proliferation within the renal sinus, renal hilum, and perirenal space, causing distortion of the pelvicalyceal system (asterisk), which is consistent with renal replacement lipomatosis. **(B–D)** Coronal and sagittal reformatted CT urography images demonstrate a widely separated pelvicalyceal system with calyceal stretching and distortion, mild wall thickening (open arrow), infundibular stenosis (dotted arrow), and calyceal narrowing (open arrowhead), characteristic imaging features of renal tuberculosis Annotations: Arrow = asymmetric parenchymal thinning; arrowhead = parenchymal calcifications; open arrow = pelvicalyceal distortion with urothelial wall thickening; dashed arrow = infundibular stenosis; open arrowhead = calyceal narrowing; asterisk = renal replacement lipomatosis.

**Figure 2:**
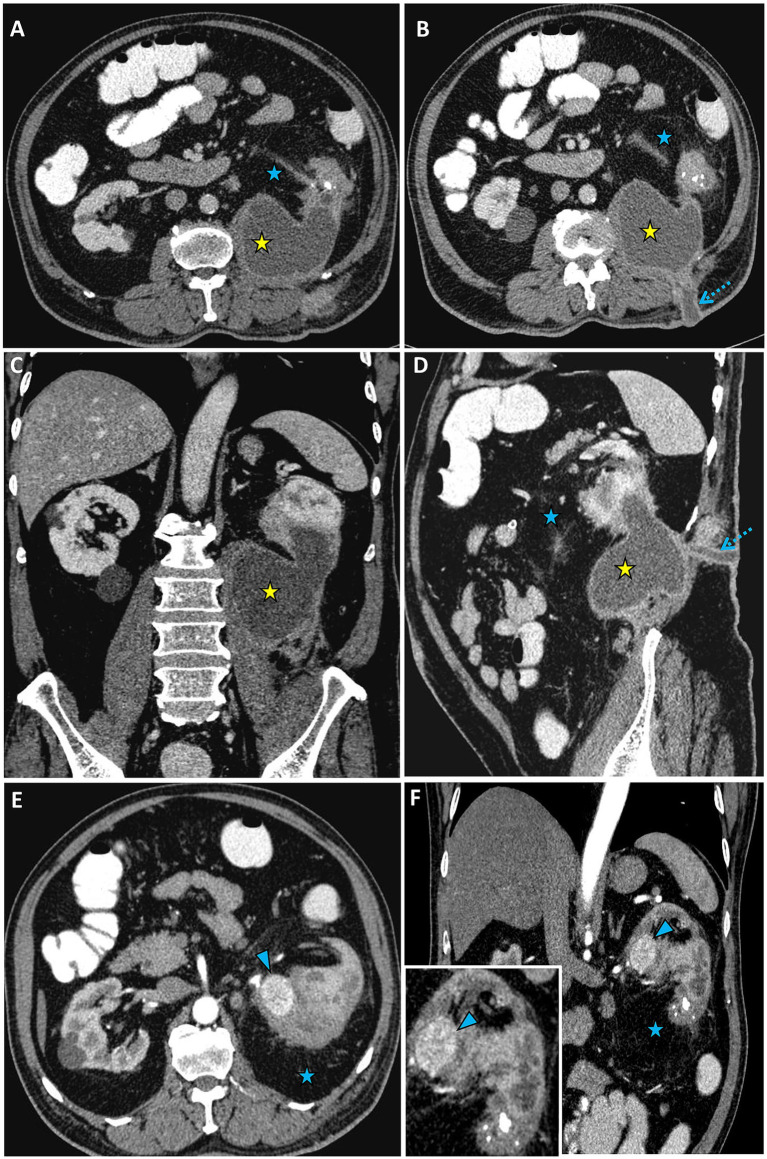
Contrast-enhanced CT demonstrating perinephric abscess, nephrocutaneous fistula, and renal cell carcinoma. **(A**–**D):** Axial **(A,B)**, coronal **(C)**, and sagittal **(D)** contrast-enhanced CT images demonstrate a large peripherally enhancing hypodense collection arising from the inferior pole of the left kidney and extending into the left psoas muscle (yellow asterisk), consistent with a perinephric abscess (dimensions: 10.0 × 6.4 × 7.5 cm) with extension into the left psoas muscle. **(B,D)**: A thin sinus tract (dotted arrow) is noted, extending from the posterior aspect of the collection through the quadratus lumborum muscle to the cutaneous surface, consistent with a nephrocutaneous fistula. **(E,F):** Axial and coronal contrast-enhanced CT images demonstrate a well-defined hyperenhancing mass in the superior pole of the left kidney (solid arrowhead), measuring 3.1 × 2.9 cm, consistent with renal cell carcinoma. Inset in panel **F**: Magnified view of the enhancing upper-pole renal mass highlights its heterogeneous enhancement and margins. Extensive fatty proliferation within the renal sinus, hilum, and perinephric region is also noted, consistent with renal replacement lipomatosis indicated by a blue asterisk. Annotations: Yellow asterisk = perinephric abscess with left psoas extension; dotted arrow = nephrocutaneous fistulous tract; arrowhead = hyperenhancing renal mass (renal cell carcinoma); blue asterisk = renal replacement lipomatosis.

Given the history of diabetes mellitus, renal calculi, and recurrent urinary tract infections, chronic pyelonephritis and xanthogranulomatous pyelonephritis were initially considered differential diagnoses. Imaging findings, including calyceal distortion and infundibular stenosis, further increased the suspicion of RTB.

### Therapeutic intervention

Due to multiple concurrent pathologies, including malignancy, a left radical nephrectomy with excision of the nephrocutaneous sinus tract was performed. The postoperative course was uneventful.

### Histopathology

Histopathological examination revealed findings that correlated with the CT findings observed in the nephrectomy specimen. The extensive sinus, hilar, and perinephric fatty proliferation identified on imaging corresponded to renal replacement lipomatosis on gross pathological evaluation. The well-defined, hyperenhancing lesion in the superior pole on CT correlated microscopically with clear cell renal cell carcinoma confined to the kidney (pT1a).

The remaining renal parenchyma exhibited multiple gray–yellow nodules and a cavity containing necrotic debris, reflecting chronic granulomatous inflammation with caseating necrosis, composed of epithelioid granulomas and multinucleated giant cells. Acid-fast bacilli staining was positive, confirming the diagnosis of renal tuberculosis. The adjacent renal parenchyma exhibited histological features consistent with chronic pyelonephritis. The excised sinus tract was lined with granulation tissue and showed chronic inflammatory changes (Figure 3).

**Figure 3:**
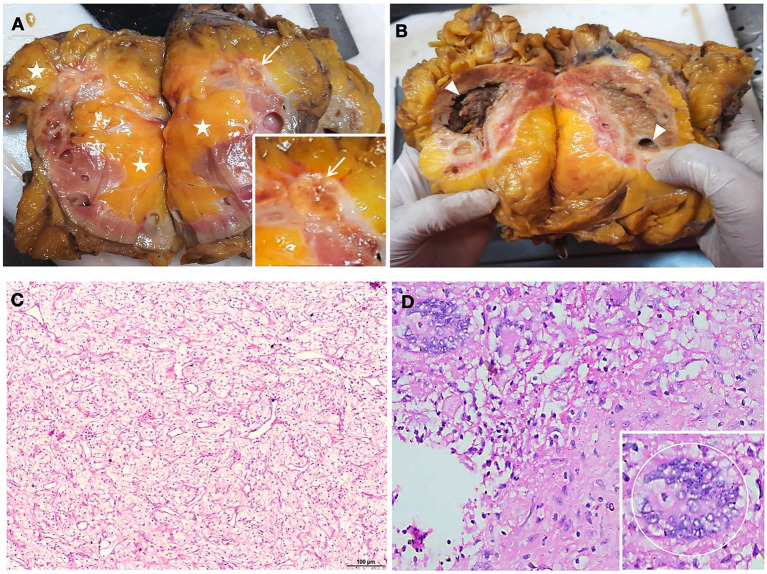
Gross and microscopic pathological findings of renal replacement lipomatosis with coexisting clear cell renal cell carcinoma and renal tuberculosis. **A**: Gross nephrectomy specimen demonstrates extensive fatty replacement of the renal hilum and renal sinus (asterisks), consistent with renal replacement lipomatosis and a gray–yellow tumor involving the superior pole (white arrows and inset). **B**: Cut section of the nephrectomy specimen demonstrates abscess and multiple sinus cavities associated with renal tuberculosis (white arrowheads). **C:** Hematoxylin and eosin (H&E)-stained section of the superior-pole lesion shows nests and sheets of clear cells separated by delicate fibrovascular septa, which is consistent with clear cell renal cell carcinoma, World Health Organization (WHO)/International Society of Urological Pathology (ISUP) Grade 2 (scale bar = 100 μm). **D**: Hematoxylin and eosin (H&E)-stained section demonstrates chronic granulomatous inflammation with epithelioid cell granulomas, central areas of caseous necrosis, and multinucleated giant cells, which is consistent with renal tuberculosis (H&E, original magnification ×200). Inset: Higher-magnification view highlighting a multinucleated giant cell (circle). Annotations: Asterisks indicate renal replacement lipomatosis; arrows indicate renal cell carcinoma; arrowheads indicate the abscess and tuberculous sinus cavities; the inset circle highlights a multinucleated giant cell.

### Follow-up and outcomes

The patient was initiated on standard ATT, consisting of a 2-month intensive phase with isoniazid, rifampicin, pyrazinamide, and ethambutol, followed by a 4-month continuation phase with isoniazid and rifampicin. The patient underwent regular follow-up for monitoring renal function, drug-related adverse effects, surveillance for RCC recurrence, and assessment of the response to ATT. The patient reported complete resolution of the lumbar sinus discharge and flank pain following surgery and completion of ATT. During follow-up, he remained clinically well with stable renal function and expressed satisfaction with the overall treatment outcome. The timeline of clinical events is summarized in Figure 4.

**Figure 4:**
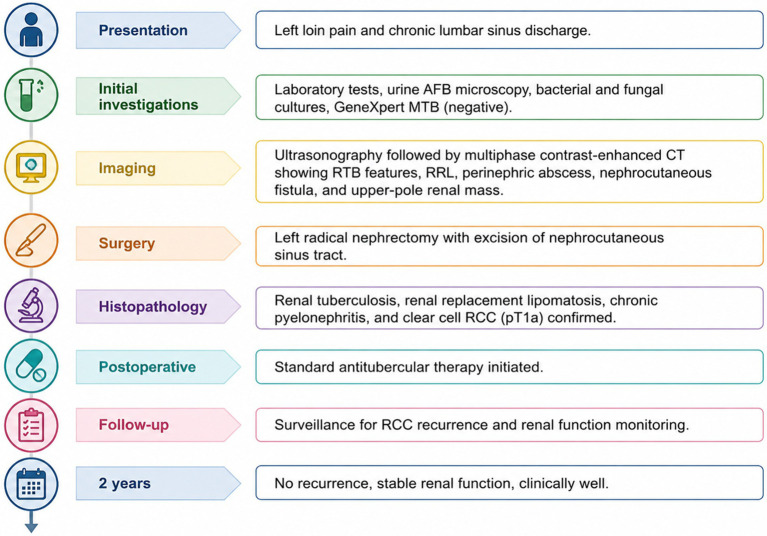
Timeline of clinical events from presentation to 2-year follow-up.

## Discussion

Renal tuberculosis (RTB) is a form of extrapulmonary tuberculosis caused by *Mycobacterium tuberculosis* and remains an important clinical entity, particularly in developing countries such as India. Extrapulmonary tuberculosis accounts for approximately 20–27% of cases worldwide and nearly 18% of cases in India (2, 5).

RTB is widely regarded as the “great masquerader” owing to its diverse and non-specific clinical and imaging manifestations, frequently resulting in misdiagnosis and delayed diagnosis until advanced stages. The disease exhibits a broad pathological spectrum, ranging from microscopic granulomas and papillary necrosis to chronic pyelonephritis, abscess formation, strictures, end-stage renal disease, and autonephrectomy, often becoming clinically apparent only after substantial renal parenchymal destruction has occurred. Immunosuppressive states and underlying medical conditions, such as diabetes mellitus and malnutrition, are recognized risk factors for renal tuberculosis (6). In our patient, diabetes mellitus and a history of calculous disease with recurrent urinary tract infections may have contributed to chronic renal inflammation and obstruction, creating a milieu conducive to persistent infection and delayed recognition of RTB.

The clinical presentation of renal tuberculosis is often insidious and non-specific, with patients commonly presenting with dysuria, frequency, flank pain, hematuria, sterile pyuria, or recurrent urinary tract infections. Delayed diagnosis may result in progressive renal parenchymal destruction and a wide range of complications.

Perinephric abscesses most commonly occur secondary to chronic pyelonephritis or xanthogranulomatous pyelonephritis, typically caused by organisms such as *Staphylococcus aureus* and *Escherichia coli*, whereas tuberculosis is an uncommon etiology. Extension into the psoas muscle with nephrocutaneous fistula formation reflects advanced renal disease with extensive parenchymal destruction and should prompt consideration of genitourinary tuberculosis, particularly in endemic regions (7, 8). In this case, these findings were consistent with an underlying chronic tuberculous process.

Renal replacement lipomatosis is a rare benign condition characterized by significant renal parenchymal atrophy accompanied by extensive fatty proliferation within the renal sinus, hilum, and perinephric space, typically occurring secondary to long-standing inflammation, obstruction, or calculous disease. Although RRL itself does not require specific treatment, it may complicate radiological interpretation by causing renal architectural distortion and influencing surgical planning and decision-making (9).

In addition to the inflammatory and degenerative changes associated with RTB and RRL, the presence of a synchronous renal mass introduced further diagnostic complexity. The coexistence of RTB and RCC within the same kidney is rare but well documented in the literature, with reported associations ranging from incidental RCC in nephrectomy specimens removed for tuberculosis to the detection of tuberculosis in the renal parenchyma adjacent to RCC, and, more rarely, the occurrence of RCC arising in a chronically non-functioning tuberculous kidney. However, current evidence does not support a clear causal relationship between the two entities.

Several hypotheses have been proposed to explain this coexistence. Chronic tuberculous inflammation may create a pro-carcinogenic microenvironment through persistent tissue injury, oxidative stress, extracellular matrix remodeling, and dysregulated tissue repair, potentially leading to metaplasia, dysplasia, and ultimately neoplastic transformation. This process may be further amplified by comorbities such as diabetes and hypertension (10, 11). Conversely, cancer-associated immune dysregulation may promote reactivation of latent tuberculosis or increase susceptibility to active infection, while tumor-derived cytokines may alter the local granulomatous milieu and facilitate the persistence of mycobacterial infection (12, 13). These mechanisms remain speculative, and this case cannot establish a temporal or causal relationship. Figure 5 schematically summarizes the proposed mechanisms linking RTB and RCC with the established pathophysiological progression and complications of renal tuberculosis.

**Figure 5:**
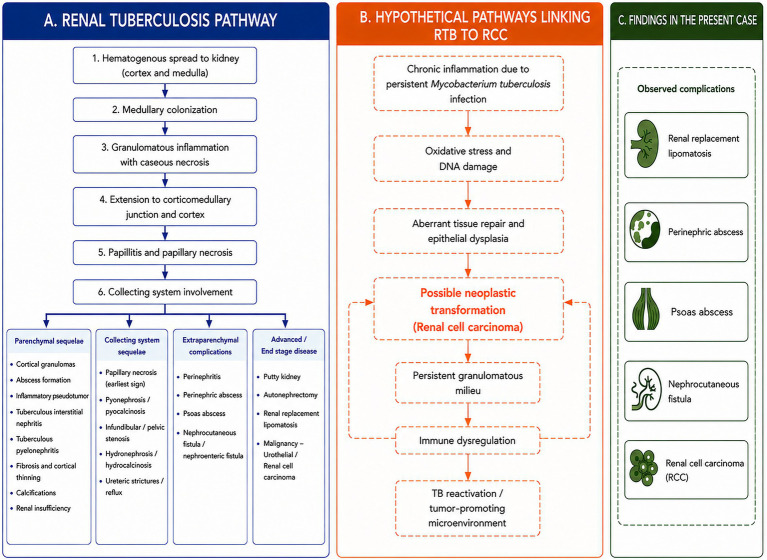
Schematic representation of renal tuberculosis pathophysiology and its complications. **(A)** Established renal tuberculosis pathway and associated complications. **(B)** Hypothetical pathways linking chronic inflammation to renal cell carcinoma development. **(C)** Complications observed in this case.

Feeney et al. (14) have highlighted the rarity of concurrent RCC and RTB, a finding further supported by a nationwide cohort study from Taiwan that demonstrated a significant association between urinary tuberculosis and urothelial carcinoma but not with RCC, suggesting that any oncogenic association may be stronger for urothelial malignancies than for RCC (15). Conde Redondo et al. (16) have described a patient with a preoperative diagnosis of renal tuberculosis in whom RCC was identified only after nephrectomy. Similarly, Peyromaure et al. (17) and Saadi et al. (18) have reported incidental renal tuberculosis in nephrectomy specimens removed for renal tumors. However, Aubert et al. (19) have described RCC developing three decades after tuberculosis in a chronically non-functioning tuberculous kidney.

Collectively, these reports highlight the diagnostic overlap between RTB and RCC, rendering preoperative recognition of their coexistence challenging and limiting standardized recommendations for diagnostic evaluation, surgical planning, and management. Unlike previously reported cases, our patient demonstrated the exceptional coexistence of isolated RTB, RRL, nephrocutaneous fistula, perinephric and iliopsoas abscesses, and synchronous clear cell RCC within a single kidney. The presence of long-standing renal calculi, recurrent urinary tract infections, and delayed diagnosis likely contributed to progressive renal destruction, locoregional extension, and the development of these advanced complications.

### Diagnostic challenges

Preoperative microbiological confirmation of RTB remains challenging because genitourinary tuberculosis is typically paucibacillary and characterized by intermittent bacilluria. Consequently, urine AFB smears, mycobacterial cultures, and nucleic acid amplification tests such as GeneXpert MTB may yield negative results despite active disease (20, 21). CT plays a pivotal role in evaluating disease extent, associated complications, and coexisting renal masses; however, advanced RTB may present as an inflammatory pseudotumor that closely mimics RCC, often necessitating histopathological confirmation (22). In this case, extensive renal destruction, together with distortion of the normal renal architecture by renal replacement lipomatosis, further complicated radiological interpretation, and the definitive diagnosis was established only after histopathological examination of the nephrectomy specimen. This case highlights the importance of integrating clinical suspicion, multimodality imaging, microbiological investigations, and histopathological examination when evaluating complex renal lesions in tuberculosis-endemic regions.

Management of this complex constellation of pathologies required a comprehensive multidisciplinary approach. The localized RCC warranted radical nephrectomy, while the tuberculous component, including the nephrocutaneous fistula, required complete excision followed by postoperative ATT. Concurrent optimization of diabetes mellitus and hypertension was essential to preserve residual renal function and facilitate postoperative recovery.

### Long-term follow-up considerations

The coexistence of RCC and RTB necessitates long-term follow-up addressing oncologic surveillance, tuberculosis recurrence, and maintenance of renal function. Oncologic surveillance following nephrectomy should be individualized according to pathological stage and recurrence risk and should include periodic clinical assessment, renal function monitoring, and cross-sectional imaging to detect local recurrence, contralateral renal involvement, or distant metastases (23).

Patients with concomitant RTB should also be monitored for disease reactivation through clinical assessment for constitutional or urinary symptoms, with microbiological testing and imaging performed when clinically indicated. Any suspicious findings should prompt microbiological confirmation and further imaging evaluation. Long-term assessment of renal function is particularly important in nephrectomized patients. Periodic monitoring of serum creatinine, estimated glomerular filtration rate, blood pressure, and contralateral kidney function is essential, as chronic kidney disease may develop despite favorable outcomes in the majority of patients with a normal remaining kidney (24).

In this case, the RCC was confined to the left kidney, and follow-up imaging performed at 6 months, 12 months, and annually thereafter demonstrated no evidence of recurrence over a 2-year period. Renal function remained stable with regular clinical and biochemical monitoring. Overall, appropriate long-term surveillance is essential to ensure favorable oncologic, infectious, and functional outcomes.

The strengths of this report include comprehensive multimodality imaging, which facilitated the recognition of multiple coexisting renal pathologies, and definitive radiological–pathological correlation following nephrectomy. Detailed histopathological examination was pivotal in establishing the rare coexistence of renal tuberculosis, renal replacement lipomatosis, and clear cell RCC within a single kidney. Limitations of this report include the lack of preoperative microbiological confirmation of RTB, indicating the limited sensitivity of urinary investigations in paucibacillary disease, the inability to establish a temporal or causal relationship between RTB and RCC, and the limited generalizability inherent to a single-case report.

## Conclusion

Isolated renal tuberculosis remains a diagnostic challenge because of its insidious course and diverse clinical and radiological manifestations. This case illustrates the rare coexistence of infectious, inflammatory, degenerative, and neoplastic renal pathologies, emphasizing the importance of multidisciplinary collaboration among urologists, radiologists, pathologists, microbiologists, and infectious disease physicians to facilitate accurate diagnosis and optimal management. Early recognition and timely initiation of antitubercular therapy remain crucial to prevent irreversible renal damage and improve patient outcomes.

## Data Availability

The original contributions presented in the study are included in the article/supplementary material; further inquiries can be directed to the corresponding author.
